# Lung Ultrasonography Scores in Preterm Infants and Respiratory Outcomes at Age 2 Years

**DOI:** 10.1001/jamanetworkopen.2024.15513

**Published:** 2024-06-07

**Authors:** Luca Bonadies, Daniele De Luca, Maria Auciello, Laura Moschino, Sabrina Congedi, Daniel Nardo, Eugenio Baraldi

**Affiliations:** 1 Neonatal Intensive Care Unit, Department of Woman’s and Child’s Health, Institute of Pediatric Research, University of Padova, Italy; 2 Division of Pediatrics and Neonatal Critical Care, A. Béclère Medical Center, Paris Saclay University Hospitals, Paris, France

## Abstract

This cohort study examines the role of lung ultrasonography score in estimating respiratory episodes needing drugs or hospitalization in premature infants.

## Introduction

Poor respiratory outcomes and frequent respiratory exacerbations are common complications of preterm birth and bronchopulmonary dysplasia (BPD).^1^ Growing evidence supports the use of lung ultrasonography score (LUS)^2^ to estimate BPD risk,^3^ but the association between LUS and later respiratory outcomes is unknown.

## Methods

Between September 2019 and November 2022, we enrolled preterm infants (<30 weeks’ gestation) to investigate the association between LUS^2^ within the first 3, 7, and 14 days of life (DOL) and respiratory outcomes (respiratory exacerbations, physician-diagnosed wheezing episodes, and respiratory medication use) at 2 years’ corrected age. Clinical data and validated respiratory morbidity score^4^ were collected. We evaluated LUS as a risk factor for respiratory outcomes, applying area under the receiver operating characteristic curve (AUC) analysis. The Province of Padua Ethics Committee for Clinical Trials approved this cohort study. Parents provided written informed consent. We followed the STROBE reporting guideline.

Two-sided *P* < .05 indicated statistical significance. Data were analyzed with IBM SPSS Statistics 28.0.1 (IBM). The eMethods in Supplement 1 provides study details.

## Results

We enrolled 51 patients (mean [SD] gestational age [GA], 27.3 (1.7) weeks; 20 females [39.2%], 31 males [60.8%]; mean [SD] birth weight: 947 [301] grams), of whom 16 (31%) developed moderate to severe BPD and 8 (16%) mild BPD. After initial discharge, 32 infants (63%) received at least 1 respiratory drug (bronchodilators, corticosteroids, and/or antibiotics), 16 (31%) were hospitalized for respiratory problems, and 3 (3%) needed pediatric intensive care unit (ICU) admission. Mean (SD) respiratory morbidity score at 2 years was 0.51 (0.76).

Compared with infants not receiving drugs or hospitalization, infants needing respiratory drugs had higher median (IQR) LUS at DOL 3 (1 [0-3] vs 6 [3-10]; *P* = .02), 7 (0 [0-1] vs 5 [2-10]; *P* < .001), and 14 (1 [0-2] vs 6 [1-11]; *P* < .001). Among infants needing hospitalization, LUS was higher at DOL 3 (3 [0-7] vs 4 [3-12]; *P* < .001), DOL 7 (1 [0-4] vs 10 [5-12]; *P* < .001), and DOL 14 (2 [0-3] vs 8 [7-12]; *P* < .001). Infants needing ICU always had LUS over 8.

At DOL 3, 7, and 14, LUS correlated with number of respiratory exacerbations needing drugs (eg, DOL 7: ρ = 0.762) or hospitalization (ρ = 0.663) and with respiratory morbidity score (ρ = 0.714), and after correction for GA for DOL 7 and 14 (eg, needing drugs: adjusted β = 0.352 and 0.177) (Table 1). LUS was associated with respiratory outcomes (eg, DOL 7 AUC, 0.838 [needing drugs] and 0.868 [needing hospitalizations]) (Table 2).

**Table 1.** Correlation of Lung Ultrasonography Score With Any Need for Respiratory Drugs, Any Need for Respiratory Hospitalizations, and Respiratory Morbidity Score

DOL	Any need for respiratory drugs				Any need for respiratory hospitalizations				Respiratory Morbidity Score			
	ρ (95% CI)	*P* value	Adjusted β (95% CI)	*P* value	ρ (95% CI)	*P* value	Adjusted β (95% CI)	*P* value	ρ (95% CI)	*P* value	Adjusted β (95% CI)	*P* value
3	0.521 (0.252-0.703)	<.001	0.079 (−0.018-0.176)	.11	0.351 (0.059-0.588)	.02	0.039 (−0.009-0.087)	.11	0.633 (0.414-0.777)	<.001	0.103 (0.063-0.143)	<.001
7	0.762 (0.609-0.861)	<.001	0.352 (0.246-0.458)	<.001	0.663 (0.465-0.798)	<.001	0.164 (0.098-0.230)	<.001	0.714 (0.536-0.826)	<.001	0.133 (0.096-0.170)	<.001
14	0.691 (0.507-0.815)	<.001	0.177 (0.068-0.285)	.02	0.535 (0.297-0.711)	<.001	0.080 (0.018-0.143)	.01	0.621 (0.404-0.767)	<.001	0.104 (0.065-0.143)	<.001

Abbreviations: Adjusted β, adjusted for gestational age β coefficient; DOL, day of life; ρ, rho coefficient.

**Table 2.** Area Under the Receiver Operating Characteristic Curve for Each Evaluated Time Point Lung Ultrasonography Score to Estimate the Risk of Any Need for Respiratory Medications and Any Need for Respiratory Hospitalizations

DOL	Any need for respiratory drugs							Any need for respiratory hospitalizations						
	AUC (95% CI)	*P* value	% (95% CI)		Best cutoff	PPV (95% CI)	NPV (95% CI)	AUC (95% CI)	*P* value	% (95% CI)		Best cutoff	PPV (95% CI)	NPV (95% CI)
			Sensitivity	Specificity						Sensitivity	Specificity			
3	0.780 (0.641-0.918)	.002	70.4 (51.3-86.8)	77.8 (52.4-93.6)	4	0.83 (0.67-0.92)	0.63 (0.48-0.77)	0.725 (0.566-0.883)	.005	84.6 (54.5-98.1)	50.0 (30.8-66.5)	3	0.37 (0.30-0.49)	0.81 (0.68-0.96)
7	0.838 (0.722-0.954)	<.001	66.7 (47.2-82.71)	77.8 (54.4-93.9)	3	0.86 (0.66-0.92)	0.57 (0.46-0.72)	0.868 (0.759-0.976)	<.001	76.9 (51.9-95.7)	78.1 (62.1-91.3)	5	0.71 (0.49-0.80)	0.85 (0.76-0.96)
14	0.792 (0.661-0.923)	.001	66.7 (47.2-82.7)	94.6 (72.7-99.8)	3	0.94 (0.74-0.99)	0.58 (0.50-0.74)	0.813 (0.658-0.967)	<.001	76.9 (51.9-95.7)	87.5 (71.8-96.6)	7	0.73 (0.54-0.88)	0.87 (0.77-0.96)

Abbreviations: AUC, area under the receiver operating characteristic curve; DOL, day of life; NPV, negative predictive value; PPV, positive predictive value.

## Discussion

Respiratory outcomes in the first 2 years of life are important clinically among premature infants with BPD.^5^ Our findings suggest that infants with later respiratory problems show signs of worse lung aeration measured by LUS in the first 14 DOL.

Significant correlation results after GA correction have been obtained as early as DOL 7, suggesting that a higher LUS at such time points is a risk factor for more frequent respiratory episodes. Different cutoff values have also been identified (higher at later time points), suggesting the presence of an active evolving disease in the lungs toward a higher LUS in infants later affected. Lung ultrasonography performed at DOL 3 may not be associated with long-term outcomes because LUS could be altered by other pathological processes, such as respiratory distress syndrome and patent ductus arteriosus. Good timing to estimate respiratory outcomes seems to be the second week of life, although the ideal DOL remains unknown.

Study limitations are the small sample and inclusion of infants born at 28 to 29 weeks’ gestation with potentially lower risk for respiratory complications. In very preterm neonates, the association of LUS, especially at DOL 7 and 14, with the number of respiratory episodes needing medications and/or hospitalization and with respiratory morbidity score suggests that LUS is a promising tool for estimating respiratory outcomes in the first 2 years of life.

## Notes

Supplement 1.
eMethods.

eReferences

Supplement 2.
Data Sharing Statement
