# Supplementary material for: Lung Ultrasonography Scores in Preterm Infants and Respiratory Outcomes at Age 2 Years
Source: JAMA Netw Open. 2024 Jun 7;7(6):e2415513. doi: 10.1001/jamanetworkopen.2024.15513 (PMC11161840; doi:10.1001/jamanetworkopen.2024.15513)
Supplement: Supplement 1. — eMethods. eReferences [file jamanetwopen-e2415513-s001.pdf]

Supplemental Online Content

Bonadies L, De Luca D, Auciello M, et al. Lung ultrasonography scores in preterm infants and respiratory outcomes at age 2 years. *JAMA Netw Open*. 2024;7(6):e2415513. doi:10.1001/jamanetworkopen.2024.15513

eMethods.

eReferences

This supplemental material has been provided by the authors to give readers additional information about their work.

© 2024 Bonadies L et al. *JAMA Network Open*

eMethods.

Study design and patients

This was a longitudinal, prospective cohort study conducted in an academic level IV NICU between September 2019 and November 2022 (enrollment ended in August 2020). The study was grated ethical approval (n° 0070403/AOPD) and written parental consent was obtained. Relevant privacy regulations and STROBE guidelines were followed. Neonates with a gestational age (GA) < 30 weeks were eligible. Patients presenting with any of the following a priori exclusion criteria were excluded: 1) enrolment in interventional studies, 2) chromosome disorders, 3) major congenital malformations, 4) massive airleaks preventing a complete ultrasound examination of the lung parenchyma; 5) pulmonary hypoplasia, or any congenital lung disorder; 6) early death. Optimal perinatal and respiratory care was provided according to the best available evidence and local protocols.

Lung ultrasound procedure

Ultrasound examinations were performed on each patient on DOL3, 7, and 14 using a portable ultrasound system (Sonosite SII (Fujifilm®, Tokio, Japan)) and a high-resolution linear 13 MHz probe. The gain was automatically adjusted, the focus was placed on the pleural line and harmonics were off. The semiquantitative LUS proposed by Brat et al. [1] was calculated. In detail, each lung was divided into three areas (upper anterior, lower anterior and lateral), and a score of 0 to 3 was assigned to each area. Each scan was performed when the infant had been supine for at least 1 hour, after airway suction, at the moment of nursing care in a quiet state. The ultrasound exams were performed and interpreted by an expert neonatologist with 3 years of LU experience, or by a specifically trained pediatric resident with experience of more than 50 supervised LU exams. Lung ultrasound findings and LUS were recorded in a dedicated spreadsheet that was not included in the patient records, so attending clinicians were blinded to these findings. An assessment of the reliability between those performing the LU studies was not performed for the purposes of this study.

Follow-up

BPD was defined as suggested by the 2001 NICHD [2]. All enrolled infants were followed-up and underwent routine visits every 3 months until 2 years of corrected age. This constitutes the routine follow-up program for extremely preterm neonates admitted to our NICU. During these visits physicians recorded: 1) need of respiratory medications (antibiotics, corticosteroids, bronchodilators), 2) need of hospitalization due to respiratory problems. A validated respiratory morbidity score was calculated as previously described. [3] In detail, we scored 0 if there was no cough, wheezing, or respiratory drug need and 1 or 2 if there were ≤ 3 or ≥ 4 days or nights of cough, wheezing, or need of respiratory drugs, respectively, during the 12th month of life. [4] Physicians in charge of the follow-up were blinded to the results of lung ultrasound.

Outcomes and clinical definitions

The aforementioned data recorded during the follow-up were considered co-primary outcomes, while the respiratory morbidity score was chosen as secondary outcome. As per local healthcare policy, each child is assigned to a family pediatrician (FP) who is the primary referral for health-related matters. In Italy, there is a tax-funded public healthcare system with universal access, and patients do not incur in any direct costs related to primary care visits.

Respiratory medications were prescribed by the FP, based on respiratory clinical signs and NICU team advice. In detail, inhaled albuterol was administered when a child presented any of the following: prolonged expiration, wheezing, dyspnea or reduced peripheral hemoglobin O_2 saturation. Corticosteroids (inhaled, oral or endovenous depending on the clinical condition) were prescribed in case of deterioration or persistency of wheezing despite inhaled albuterol. Antibiotics were given in presence of fever, respiratory signs or increased inflammatory markers. Hospitalization was dictated based on previously published criteria [5] and infants were admitted to the pediatric intensive care unit when they needed any respiratory support beyond simple oxygen supplementation.

Statistics

Lacking of previous evidence on which we could have based our sample size choice, we decided a convenience sample size of 50 infants, as this approximately represents the number of preterm infants born below 30 weeks

© 2024 Bonadies L et al. *JAMA Network Open*

of gestation annually admitted to our prematurity follow-up service. This numerosity appears to be similar to the population recruited in the only study lately available in this field[6]. Data were reported as mean (standard deviation), median (interquartile range) and as numbers (%), as appropriate. The correlations between LUS and co-primary outcomes, and between LUS and the respiratory morbidity score were analyzed with the Spearman correlation coefficient followed by multivariate linear regression adjusted for GA as this is known to influence the long-term respiratory outcome [6].

The ability of LUS to predict long-term respiratory outcomes was analyzed with the receiver operator characteristics (ROC) analysis, evaluating sensitivity, specificity, positive predictive value and negative predictive value of the test. Best cut-off value was identified applying Youden test. Data were analyzed with IBM SPSS Statistics for Windows (ver. 28.0.1) and a p-value <0.05 was considered statistically significant.

eReferences

1. Brat R, Yousef N, Klifa R, Reynaud S, Shankar Aguilera S, De Luca D. Lung Ultrasonography Score to Evaluate Oxygenation and Surfactant Need in Neonates Treated With Continuous Positive Airway Pressure. *JAMA Pediatr*. 2015;169(8):e151797. doi:10.1001/jamapediatrics.2015.1797

2. Jobe AH, Bancalari E. Bronchopulmonary dysplasia. *Am J Respir Crit Care Med*. 2001;163(7):1723-1729. doi:10.1164/ajrccm.163.7.2011060

3. De Luca D, Shankar-Aguilera S, Autilio C, et al. Surfactant-secreted phospholipase A2 interplay and respiratory outcome in preterm neonates. *Am J Physiol Lung Cell Mol Physiol*. 2020;319(1):L95-L104. doi:10.1152/ajplung.00462.2019

4. Parad RB, Davis JM, Lo J, et al. Prediction of respiratory outcome in extremely low gestational age infants. *Neonatology*. 2015;107(4):241-248. doi:10.1159/000369878

5. Baraldi E, Lanari M, Manzoni P, et al. Inter-society consensus document on treatment and prevention of bronchiolitis in newborns and infants. *Ital J Pediatr*. 2014;40:65. doi:10.1186/1824-7288-40-65

6. Savoia M, Miletic P, De Martino M, Morassutti FR. Lung ultrasound score follows the chronic pulmonary insufficiency of prematurity trajectory in early infancy. *Eur J Pediatr*. 2022;181(12):4157-4166. doi:10.1007/s00431-022-04629-y

© 2024 Bonadies L et al. *JAMA Network Open*
