# Supplementary material for: Lung Ultrasonography Scores in Preterm Infants and Respiratory Outcomes at Age 2 Years
Source: JAMA Netw Open. 2024 Jun 7;7(6):e2415513. doi: 10.1001/jamanetworkopen.2024.15513 (PMC11161840; doi:10.1001/jamanetworkopen.2024.15513)
Supplement: Supplement 2. — Data Sharing Statement [file jamanetwopen-e2415513-s002.pdf]

Data Sharing Statement

Bonadies. Lung Ultrasonography Scores in Preterm Infants and Respiratory Outcomes at Age 2 Years. *JAMA Netw Open*. Published June 07, 2024.
doi:10.1001/jamanetworkopen.2024.15513

Data
Data available: Yes
Data types: Deidentified participant data
How to access data: Deidentified participant data will be made available upon reasonable request by the first author (luca.bonadies@unipd.it)
When available: With publication

Supporting Documents
Document types: None

Additional Information
Who can access the data: Data will be made available to researchers whose proposed use of the data has been approved
Types of analyses: E.g. meta-analysis
Mechanisms of data availability: with investigator support and after approval of a proposal
